# Biostimulant Potential of Humic Acids Extracted From an Amendment Obtained via Combination of Olive Mill Wastewaters (OMW) and a Pre-treated Organic Material Derived From Municipal Solid Waste (MSW)

**DOI:** 10.3389/fpls.2018.01028

**Published:** 2018-07-20

**Authors:** Giuseppe Palumbo, Michela Schiavon, Serenella Nardi, Andrea Ertani, Giuseppe Celano, Claudio M. Colombo

**Affiliations:** ^1^Dipartimento di Agricoltura, Ambiente e Alimenti, Università degli Studi del Molise, Campobasso, Italy; ^2^Dipartimento di Agronomia, Animali, Alimenti, Risorse Naturali e Ambiente, Università di Padova, Legnaro, Italy; ^3^Dipartimento di Farmacia, Università degli Studi di Salerno, Fisciano, Italy

**Keywords:** *Zea Mays* L., bio-oxidation, amendments, humic acids, biostimulants, FT-IR, nitrogen assimilation, glycolysis

## Abstract

Olive mill wastewaters (OMW) contain significant levels of phenolic compounds with antimicrobial/phytotoxic activity and high amounts of undecomposed organic matter that may exert negative effects on soil biology. Among OMW detoxification techniques, those focusing on oxidative degradation of phenolic compounds are relevant. The composting (bio-oxidation) process in particular, exploits exothermic oxidation reactions by microorganisms to transform the organic matrix of OMW into an amendment biologically stable and feasible to use in agriculture. This process consists of an active phase during which organic compounds are rapidly decomposed, and a curing phase characterized by a slow breakdown of the remaining materials with the formation of humic substances (HS) as by-products. In this study, bio-oxidation of OMW was performed using a pre-treated organic material derived from municipal solid waste (MSW). The obtained amendment (OMWF) was stable and in accordance with the legislative parameters of mixed organic amendments. HS were then extracted from OMWF and MSW (control amendment, Amd-C), and differences in structural properties of their humic acid (HA) fraction were highlighted via spectroscopy (Fourier Transform Infrared) and Dynamic Light Scattering. To assay a potential use of HA as biostimulants for crops, 12-day old *Zea Mays* L. plants were supplied with HA at 0.5 mg and 1 mg C L^-1^ for 2 days. HA from both amendments increased plant growth, but HA from OMWF was more effective at both dosages (plus 35–37%). Also, HA from OMWF enhanced both nitrogen assimilation and glycolysis by increasing the activity of nitrate reductase (∼1.8–1.9 fold), phosphoglucose isomerase (PGI) (∼1.8–2 fold) and pyruvate kinase (PK) (∼1.5–1.8 fold), while HA from Amd-C targeted glycolysis preferentially. HA from OMWF, however, significantly stimulated plant nutrition only at lower dosage, perhaps because certain undetermined compounds from detoxified OMW and incorporated in HA altered the root membrane permeability, thus preventing the increase of nutrient uptake. Conversely, HA from Amd-C increased nutrient accumulation in maize at both dosages. In conclusion, our results indicate that the amendment obtained via OMW composting using MSW had a reduced pollution load in terms of phenolic compounds, and HA extracted from OMWF could be used as valuable biostimulants during maize cultivation.

## Introduction

Olive mill wastewaters (OMW), also named olive vegetable waters, are endowed with properties that depend on the fruit variety and maturity, climate, soil type and extraction procedure (Borja et al., 2006). The disposal of OMW poses a concern to the olive oil industry, which is widely developed in the Mediterranean countries, such as Spain, Italy, Greece, and Tunisia (Niaounakis and Halvadakis, 2006; International Olive Council, 2014; Koutsos et al., 2018). These countries produce on average 2.74 million tons of olive oil per year, which account for 98% of the world production (Ahmadi-Esfahani, 2006). Italy is the second largest olive oil producer in the European Union (EU), with an estimated olive oil production of 500–600 thousand tons per year (Incelli et al., 2016).

The oil extraction process requires water in large amounts and generates an annual world production of wastewaters to be treated as high as 30 million tons (Roig et al., 2006; Mechri et al., 2007; Sellami et al., 2008). OMW are characterized by a high pollutant load because of their significant content in phenolic compounds with antimicrobial-phytotoxic action and limited biodegradability (Komilis et al., 2005; Justino et al., 2012). They also contain significant amounts of undecomposed organic substance, which may exert negative effects on soil biology and properties (Di Serio et al., 2008; Ntougias et al., 2013).

Unfortunately, a common European legislation framework concerning the management and the recycling of OMW in agriculture does not still exist. Therefore, EU countries discretely set the threshold limit values for the safe OMW disposal and re-use (Koutsos et al., 2018). In Italy, the disposal of OMW as amendments in agriculture is currently regulated by Law 574/96, which allows the shedding of OMW on soil within the range of 50–80 m^3^ ha^-1^. Spreading practices of OMW on soil exceeding this threshold might be harmful to the ecosystem and cause failure of crop cultivation (Ouzounidou et al., 2010; Incelli et al., 2016). However, whether properly detoxified, OMW could be used as organic fertilizers to improve soil fertility under conditions of nutrient and organic matter shortage (Mekki et al., 2006; Nasini et al., 2013). Poor and unstructured soils for instance, can be enriched in nitrogen (N), phosphorus (P), and potassium (K) through spreading of detoxified OMW (Isidori et al., 2005).

Olive mill wastewaters detoxification techniques include treatments focused on the degradation of bioactive molecules, primarily phenolic compounds (Casa et al., 2003; Silva et al., 2007). Among these techniques, the most widely employed are those based on flocculation/coagulation in water (Khoufi et al., 2008; Flores et al., 2018), ozonization (Andreozzi et al., 1998), centrifugation and ultrafiltration (Arvanitoyannis et al., 2007), sonication (Oztekin and Sponza, 2013), anaerobic digestion, dilution (Hamdi et al., 1991; Andreozzi et al., 2008; El-Gohary et al., 2009), oxidative degradation via addition of manganese and iron oxides (Colarieti et al., 2006), and composting or bio-oxidation (Paredes et al., 2002; Sanchez Monedero et al., 2002; Sampedro et al., 2007). This last approach in particular, exploits exothermic oxidation reactions by microorganisms to transform the organic matrix of OMW into a biologically stable and odorless amendment, feasible to use in agriculture (Ghanbari et al., 2012; Gigliotti et al., 2012). The quality of the amendment is evaluated based on the absence of pathogens and heavy metals.

Specifically, the composting process consists of two stages in sequence: an active, thermophilic phase, during which organic components undergo intense and rapid degradation activity and the break down of phytotoxic compounds occurs and proceed until the biological stability of the process is achieved; a curing phase, characterized by the degradation and further transformation of recalcitrant organic components, with the formation of humic substances (HS) (Adani et al., 1997). In this process, it is possible that some of the phenolic compounds contained in OMW detoxified in this way become part of the HS instead of being degraded.

Humic substances comprise humic and fulvic acids, and consist of small molecules of amphiphilic nature able to generate molecular aggregates or supramolecular assemblies in solution and on mineral surfaces (Wershaw, 1999; Piccolo, 2001; Schaumann, 2006). HS influence plant physiology by triggering complex transcriptional networks through an intricate mechanism of action involving auxin- dependent and independent signaling pathways (Muscolo et al., 2013; Nardi et al., 2016, 2017). They are also widely recognized as biostimulants, i.e., products containing substances and/or microorganisms whose function in trivial amounts is to promote plant growth-related processes, enhance plant nutrient uptake and use efficiency, resistance and tolerance to abiotic stress, and improve the quality of crop-derived products (European Biostimulants Industry Council [EBIC], 2013). The effects of HS in plants depends on their concentration, molecular weight and physical-chemical properties. Strong evidence exists that HS effects in plants are in part due to their content in substances displaying hormone-like activity (Nardi et al., 1994; Mora et al., 2010). HS can stimulate plant nitrogen (N) uptake and assimilation (Varanini and Pinton, 2001; Vaccaro et al., 2009), and induce changes in root architecture, especially in the early phases of plant development (Canellas et al., 2002; Nardi et al., 2002; Zandonadi et al., 2007). Additionally, HS enhance the root H^+^-ATPase activity (Nardi et al., 1991; Zandonadi et al., 2010) and control nutrient availability in maize (Eyheraguibel et al., 2008).

Combining the importance of HS in plant productivity and the idea of recycling OMW for agricultural purposes, the aim of this study consisted in: (i) detoxifying OMW via a bio-oxidation process using a pre-treated organic material derived from municipal solid waste (MSW); (ii) extracting HA from the resulting amendment and compare their properties with those of HA obtained from a control amendment (MSW without OMW); (iii) testing whether the obtained HA displayed beneficial properties on maize (*Zea Mays* L.) plant metabolism and could be used as valuable biostimulants.

## Materials and Methods

### Chemical and Physical Analyses of OMW

Olive mill wastewaters were furnished by an olive mill farm located in Isernia (Molise region, Italy) and produced via olive oil centrifugation using a three-phase process. Three samples (100 g each) of OMW were collected and analyzed. With respect to nitrogen (N) forms, the content of ammonium (NH_4_^+^) was determined using the Nessler method reported by Trombetta et al. (1998), while NO_3_^-^ and NO_2_ were quantified via steam distillation procedures described by Bremner and Keeney (1965).

The remaining chemical analyses were performed according to the analytical procedures reported in the official methods of Italian soil chemical analysis (Violante, 2000).

For total phenol determination, 5 mL of OMW were centrifuged for 5 min at 5,000 g; 0.25 mL of supernatant were then added with 0.5 mL ethyl acetate, and the obtained extract was stirred and centrifuged for 5 min at 5,000 *g*. The extraction procedure was repeated three times with further additions of ethyl acetate (0.5 mL). Finally, the supernatant was dried at room temperature for about 48 h. The solid extract was solubilized using 0.25 mL of a mixture containing methanol and water in the ratio 4:1 (v:v), and then vortexed for 2 min. The extract was placed in 10 mL tubes, added with 1 mL of distilled H_2_O, 0.9 mL of 0.5 M NaHCO_3_ (pH 8.5), and 1 mL of diluted acetate 1/10 (v/v). The extract was then stirred for 2 min and after 2 h the content of total phenols was determined via spectrophotometer at λ = 765 nm according to the Folin–Ciocalteu method described by Zullo et al. (2014). The amount of total polyphenols was expressed in mg dm^-3^ of gallic acid. Quantification of individual phenols was performed as described below for MSW.

Analyses were all conducted on 100 g of homogenized of the same OMW.

### Chemical and Microbiological Analyses of MSW

Before being processed with OMW, the organic material derived from MSW was analyzed for the presence of microorganisms in order to exclude the existence of human pathogens, mainly enteric bacteria, viruses, protozoa, and helminthes (Bonadonna et al., 2002). Total and fecal coliforms were absent, thus attesting the good hygienic conditions of the matrix. The determination of total organic carbon, Kjeldahl nitrogen, phosphorus Olsen, electrical conductivity and pH were performed according to the methods of Violante (2000). Specifically, total organic carbon was analyzed by the method Springer-Klee, nitrogen was determined using the Kjeldahl method, phosphorus (P) Olsen was measured spectrophotometrically at λ = 720 nm, electrical conductivity and pH were measured in a sample suspension added with distilled water in the ratio 1:1 (v:v). Elemental analysis was conducted via ICP-OES (Inductive Coupled Plasma Optical Emission), after the sample was digested with a solution of 65% HNO_3_/37% HCl (ratio HNO_3_/HCl 1:3 v/v), warmed until boiling for 30 min under agitation, according to the manufacturer’s instructions, and filtered at 0.45 μm filter (Millipore). Analyses were all performed on three samples (100 g each) of MSW.

### Bio-Oxidative Process for OMW Composting

Detoxification of OMW was performed through the addition of OMW to a pre-treated organic material derived from MSW, under conditions (T = 65°C) that prevented the growth of pathogenic organisms, while favored the development of bacteria required for OMW composting. The content in polyphenols was measured after 6 months since the beginning of the bio-oxidative process.

The OMW (20 L) was gradually added to MSW to favor the adsorption process and get a ratio of 1:1 on the organic matrix. Urea (2%, v/v) was also added. The resulting amendment was named OMWF (olive mill water filter plus MSW pre-treated organic material). The MSW pre-treated organic material added with 2% (v/v) urea was used as control (Amd-C). The organic material lodged in the bins was turned over periodically, every 20 days. The composting process was monitored for 6 months and total carbon and total nitrogen were determined in the amendments using the Springer–Klee and Kjeldahl methods, respectively (Violante, 2000) in three replicates. The efficacy of the bio-oxidative process in reducing OMW toxicity was evaluated by measuring the variation in content and profile of phenolic compounds. Extraction of total polyphenols from the amendments was performed using ethyl acetate as described previously, and their content was determined via HPLC using a UV-VIS detector DAD at 280 nm and a column Synchronis C18, 15 cm in length and 4.6 cm in diameter, with a particle size diameter of 5 μm. The separation of the different fractions was performed according to the following conditions: 0 to 15 min by using a gradient mixture of 95% acetic acid 0.5% (A) and 5% acetonitrile (B) up to 80% A and 20% B, with isocratic separation up to 30 min. The identification of phenolic compounds was carried out by comparing their retention times (RT) and online UV spectra with those of reference standards corresponding to phenolic compounds commonly present in most OMW (hydroxybenzoic acid, syringic acid, verbascoside and ferulic acid). Standards were provided by Sigma-Aldrich Ltd.

### Chemical Extraction of Humic Acids (HA) From the Amendments

Humic substances extraction was carried out according to Nardi et al. (1994). Chemical fractioning in humic acids (HA), fulvic acids (FA), and humin (HU) was based on the differential solubility of the organic fractions of the amendment depending on the pH. Control (Amd-C) and OMWF amendments were placed in individual 500 mL Erlenmeyer flasks and added with 0.5 M NaOH (40 mL) and distilled water (80 mL). Nitrogen (N) in each flask was insufflated for a few seconds. The suspensions were shaken for 6 h and left to rest for further 12 h. Each suspension was then centrifuged at 6,000 × *g* in order to obtain two fractions, one containing the total extractable carbon (TEC). The TEC-containing solution was placed in a cylinder and acidified with HCl (ratio 1:1, v/v) to achieve a pH lower than 2, which allowed the separation of the HA fraction from the supernatant (FA fraction). Both HA and FA were purified and dialyzed. HA were initially freeze-dried and their content in C was measured using an elemental analyzer (Vario MACRO CNS, Hanau, Germany). Then, an amount of them corresponding to 10 mg C was re-suspended in deionized water in the presence of few drops of pure NH_3_ to obtain a HA stock solution with 1 mg ml^-1^ final concentration. NH_3_ was removed using rotavapor in the presence of acidic trap.

### FTIR Spectroscopy of Humic Acids

Before performing spectroscopic analyses, samples of HSs obtained from Amd-C and OMWF amendments were reduced to an impalpable powder in agate mortar using potassium bromide (KBr), and kept in a desiccator for 24 h with silica gel.

FTIR spectra of Amd-C and OMWF were obtained via a VERTEX70/70v high-resolution spectrophotometer (Bruker, Italy). The absorbance spectra were collected at a spectral resolution of 4 cm^-1^ with 256 scans, between 400 and 4,000 cm^-1^ and converted to absorbance using the software OPUS 6.5 (Bruker Optics).

Spectroscopic analyses were performed on three samples of HA. We only show one representative FTIR spectrum.

### Dynamic Light Scattering (DLS) of Humic Acids: Particle Size (PS) and Electrophoretic Mobility (EM)

Both PS and EM measurements were performed at 25 ± 0.1°C with a Zetasizer Nano-ZS (Malvern, Instruments), consisting of an Avalanche photodiode (APD) detector and a 4 mW He-Ne laser (λ = 633 nm). This instrument was widely used for a large variety of colloidal dispersions. ζ potential data were calculated from EM by the Henry equation (1):

(1) $$EM=\;\frac{2\varepsilon \zeta }{3\eta }f(\kappa R)$$

Where 𝜀 is the dielectric constant, η the viscosity, *R* the particle hydrodynamic radius and κR the ratio of R to Debye length. To convert EM into ζ the Smoluchowski factor *f*(κR) = 1.5 was used (valid for κR > 1). Effective voltage gradient was in the range 40–140 mV mm^-1^.

Particle size distributions and Poly Dispersity Index (PDI) were obtained from the intensity autocorrelation function by the cumulate and CONTIN methods, respectively, using the Malvern software (DTS Version 6.01). The apparent hydrodynamic diameter *D* was calculated from the *Z*-average translation diffusion coefficient through the Stokes–Einstein equation (2) assuming spherical particles:

(2) $$D=\frac{k_{\text{B}}T}{\text{3}\pi \eta D}$$

Where *k*_B_ is the Boltzmann constant, *T* is temperature, η the viscosity and *D* is the apparent hydrodynamic diameter.

Particle size and ξ data of aqueous dispersions of humic acids extracted from amendment and humic acids extracted from amendment adsorbed with vegetation waters were monitored in the pH range 2–10. Data represented the mean of three replicates.

### Plant Growth Experimental Design

*Zea mays* L. seeds (var. DKc 5783, DeKalb, Lodi, IT) were soaked in distilled water for one night and then surface-sterilized in 5% (v/v) sodium hypochlorite for 10 min while shaking (Ertani et al., 2009). The seeds were left to germinate for 60 h in the dark at 25°C on a filter paper wetted with 1 mM CaSO_4_. Germinated seedlings were transplanted into 3 L pots (density of plants = 10 per pot) equipped with net holds into the top, which provided a hydroponic floating system for plant growth. Roots floated in a modified Hoagland nutrient solution (Hoagland and Arnon, 1950) that was maintained aerated via air insufflation. The nutrient solution was renewed every 48 h and had the following composition: (μM): KH_2_PO_4_ (40), Ca (NO_3_)_2_ (200), KNO_3_ (200), MgSO_4_ (200), FeNaEDTA (10), H_3_BO_3_ (4.6), CuCl_2_^.^2H_2_O (0.036), MnCl_2_^.^4H_2_O (0.9), ZnCl_2_ (0.09), NaMoO^.^2H_2_O (0.01). Plants were cultivated in a climate chamber under 14 h light/10 h dark cycle, with an air temperature of 27/21°C, relative humidity of 70/85% and at a photon flux density of 280 mol m^-2^ s^-1^. On the 12th day, part of the plants was treated for 48 h with humic acids (HA) extracted from Amd-C and OMWF amendments. Based on their C content (28 and 26% w/w for Amd-C and OMWF, respectively), different volumes of HA from stock solutions (1 mg ml^-1^) were added to the nutrient solution in order to supply plants with two different C concentrations: 0.5 mg and 1 mg carbon per liter (C L^-1^). The HA concentrations were chosen based on previous studies where the most pronounced effects of HS in plants were observed at 0.5 mg and 1 mg C L^-1^ in a period as short as 48 h.

The remaining part of the untreated plants was used as a control (C). For each experimental condition, three pots were prepared. The experiment was repeated three times and was performed according to a randomized block design.

At the end of the 48 h, the plantlets were collected and divided in leaves and roots. Roots were carefully washed with distilled water first, and then with 10 mM EDTA for 15 min to remove any metal remained in root apoplast. Plant material was blot-dried and analyzed for dry weight and activities of marker enzymes.

For dry weight measurement, 30 plants were used (10 per treatment from each pot). Plants were divided into roots and leaves, and weighed separately. The samples were placed in a drying oven for 2 days at 70°C and allowed to cool down for 2 h inside a closed bell jar.

### Mineral-Nutrient Determination

The determination of mineral nutrients in leaves and roots of maize plants was performed after an acid-digestion procedure (HNO_3_/HCl 1:3, v/v). All digestion reactions were carried out in closed Teflon vessels of 120mL volume using 500 mg plant material and 10 mL of 30% (v/v) HCl as a solvent. Digested samples were then diluted in 10 mL ultrapure water and assayed via Inductively Coupled Plasma–Optical Emission Spectroscopy (ICP-OES, Optima 2000 DV, Perkin Elmer Instruments Germany).

### Enzyme Extraction and Assay Conditions, Protein Quantification

For enzyme activity assays, leaf material from five individual plants (biological replicates) per treatment was used.

For the extraction of phosphoglucose isomerase (PGI) and pyruvate kinase (PK) enzymes, leaves (1 g) from maize plants subjected to different treatments were ground in a mortar using liquid nitrogen and homogenized for 5 min in the presence of 100 mM HEPES-NaOH pH 7.7, 10 mM MgCl_2_^.^6H_2_O, 0.4 mM Na_2_EDTA, 100 mM Na ascorbate, 1% (w/v) polyvinylpyrrolidone (PVP), 1% (w/v) BSA (bovine serum albumin), and 5 mM reduced glutathione (GSH). The homogenate was then filtered and centrifuged at 20,000 × *g* for 20 min at 4°C.

Phosphoglucose isomerase (PGI, EC 5.3.1.9) assay: 530 μL 0.2 M Tris adjusted with 0.1 M HCl to pH 9.0 were added with 75 μL 20 mM β-NADH-Na_2_-salt in distilled water, 75 μL 80 mM fructose-6-phosphate-Na_2_ in 0.2 M Tris pH 9.0, and 20 μL glucose 6-phosphate dehydrogenase (from yeast) diluted to 30 U mL^-1^ with 0.2 M Tris pH 9.0. The reaction was started by adding 50 μL extract after a lag time of 20 min at 30°C (Nowotny et al., 1998). Measurements were performed spectrophotometrically for 60 s at λ = 340 nm.

Pyruvate kinase (PK, EC 2.7.1. 40) assay: 450 μL 0.1 M triethanolamine (TEA) adjusted with NaOH to pH 7.75 were added with 50 μL 3 mM β-NADH-Na_2_-salt in 0.1 M TEA pH 7.75, 50 μL 0.15 M MgSO_4_^.^6H_2_O and 0.15 M KCl in 0.1 M TEA (pH 7.75), 50 μL L-lactic dehydrogenase diluted to 225 U mL^-1^ with TEA (pH 7.75), and 50 μL extract. The reaction was started after a lag time of 10 min at 30°C by adding 50 μL 0.225 M 2-phosphoenolpyruvate-Na-H_2_O in 0.1 M TEA (pH 7.75) (Nowotny et al., 1998). Measurements were performed spectrophotometrically at λ = 340 nm.

For the extraction of nitrate reductase (NR, E.C.1.7.1.1), leaf tissues (1 g) were ground in a mortar and added with 100 mM HEPES-NaOH pH 7.5, 5 mM MgCl2, and 1 mM dithiothreitol (DTT). The ratio of plant material to mixture solution was 1:3 (v/v). The extract was filtered through two layers of muslin and clarified by centrifugation at 20,000 *g* × 15 min. The supernatant was then used for enzymatic analysis. All steps were carefully performed at 4°C (Schiavon et al., 2008). The activity of nitrate reductase (NR) was assayed in a solution containing 100 mM KH_2_PO_4_, 100 mM KNO_3_, and 400 mL of enzyme extract. The activity was measured spectrophotometrically at λ = 540 nm, and the calibration curve was plotted with known concentrations of NaNO_2_ (Lewis et al., 1982).

The total content of proteins was measured in leaves (1 g) from three individual plants (biological replicates) per treatment, estimated via the Bradford (1976) method and expressed in milligrams per gram of fresh weight (mg g^-1^ of fresh weight).

### Statistical Analysis

The analysis of variance (ANOVA) was performed using the SPSS software version 18.0 (SPSS, Chicago, IL, United States), and was followed by pair-wise *post hoc* analyses (Student–Newman–Keuls test) (Sokal and Rohlf, 1969) to determine which means differed significantly at *p* < 0.05 (±std).

## Results

### Chemical Properties of OMW

In **Table 1** the main chemical properties of OMW are reported. The pH value was within the typical range of OMW (4–6.7) (Incelli et al., 2016), while the chemical oxygen demand (COD) and the biological oxygen demand (BOD) displayed high values (Rinaldi et al., 2003). With respect to inorganic N species, NH_4_^+^ was prevalent over N oxidized forms (NO_3_^-^ and NO_2_). The content of the mineral fraction, in particular of potassium, phosphorus and calcium was medium–high, whereas Na^+^ was high. The amount of total suspended particles and total phenolic compounds (TPC) was elevate.

**Table 1:** Chemical properties of OMW sample.

Parameter	Values	Unit
pH	4.7 ± 0.5	
COD	70 ± 10	g dm^-3^
BOD	32.5 ± 2.5	g dm^-3^
Cl^-^	5.5 ± 0.5	mg dm^-3^
SO_4_^2-^	0.10 ± 0.02	mg dm^-3^
P Tot	180 ± 10	mg dm^-3^
NH_4_^+^	125 ± 25	mg dm^-3^
NO_3_^-^	3.5 ± 0.5	mg dm^-3^
NO_2_^-^	6.0 ± 0.6	mg dm^-3^
TPC	5.0 ± 1.0	g dm^-3^
TSP	75 ± 15	g dm^-3^
Na^+^	15 ± 2.5	mg dm^-3^
K^+^	5,000 ± 1,000	mg dm^-3^
Mg^2+^	7.5 ± 2.5	mg dm^-3^
Ca^2+^	20.0 ± 7.3	mg dm^-3^

*COD, chemical oxygen demand; BOD, biological oxygen demand; TPC, Total Phenols Compounds; TSP, Total Suspended Particles. In table, standard deviations are reported (n = 3).*

### Chemical Properties of MSW and Effects of the Bio-Oxidation Treatment on C and N Contents in the Amendments

The organic material used for the composting process was below the limit (<20% TOC) prescribed for organicibed for organic fertilizers by the Italian regulations (amendments, Italian law 748/1984), and the C/N ratio was slightly below 10. The concentration of all metals was also below the threshold of toxicity (**Table 2**).

**Table 2:** Chemical parameters of the organic material from MSW.

Parameter	Values	Unit
Dry leftover	73.0 ± 5.2	%
Moisture	26.9 ± 1.8	%
pH	7.44 ± 0.6	7.44
EC	5,600 ± 120	μS cm^-1^
CSC	46 ± 8	cmol(+) kg^-1^
TOC	161.4 ± 15.6 (<200)	g kg^-1^
HS	12.26 ± 3.5	g kg^-1^
TN	17.8 ± 2.9	g kg^-1^
C/N	9.06 ± 0.35	
P_olsen_	7.7 ± 1.4	mg kg^-1^
K_2_O	0.68 ± 0.20	g kg^-1^
Cd	0.10 ± 0.02 (<1.5)	mg kg^-1^
Cr	18.10 ± 2.14 (<0.5)*	mg kg^-1^
Hg	0.007 ± 0.001 (<1.5)	mg kg^-1^
Ni	5.04 ± 1.12 (<100)	mg kg^-1^
Pb	15.33 ± 2.10 (<140)	mg kg^-1^
Cu	30.66 ± 10.8 (<230)	mg kg^-1^
Zn	60 ± 12 (<600)	mg kg^-1^

*EC, electric conductivity; CSC, cation exchange capacity; TOC, total organic carbon; HS, humic substances; TN, total nitrogen. In table, standard deviations are reported (n = 3). In brackets, the threshold for TOC, TN, P and metals is shown. ^∗^The limit in the brackets is referred to hexavalent Cr only*.

**FIGURE 1:**
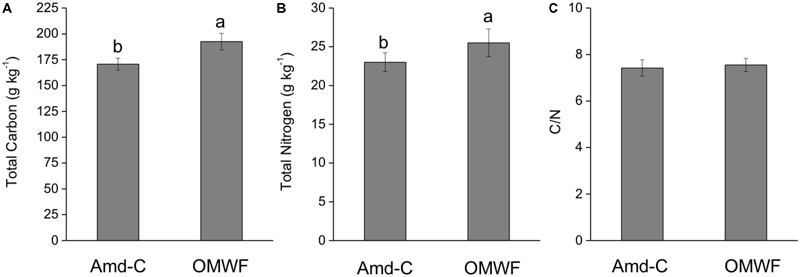
Total carbon **(A)**, total nitrogen **(B)** content, and C/N ratio **(C)** of control amendment (Amd-C) and olive mill water filter plus municipal solid waste (MSW) pre-treated organic material (OMWF) amendment. Different letters above bars indicate significant differences between treatments at *P* < 0.05 according to Student–Newman–Keuls test.

The weekly monitoring of both temperature and matrix water content followed the organic material maturation. After 6 months since the beginning of the bio-oxidation process, a higher content in C (plus 13%) and N (plus 11%) was observed in OMWF in comparison with Amd-C (**Figures 1A,B**). Because the increases in C and N were comparable, the C/N ratio was not significantly influenced by the additions of the OMW (**Figure 1C**). Generally, an ideal amendment should have a C/N ratio equal to 10, which is required for the normal microbial growth and the optimal process of humification.

### Effect of the Bio-Oxidation Treatment on OMW Phenolic Content

**FIGURE 2:**
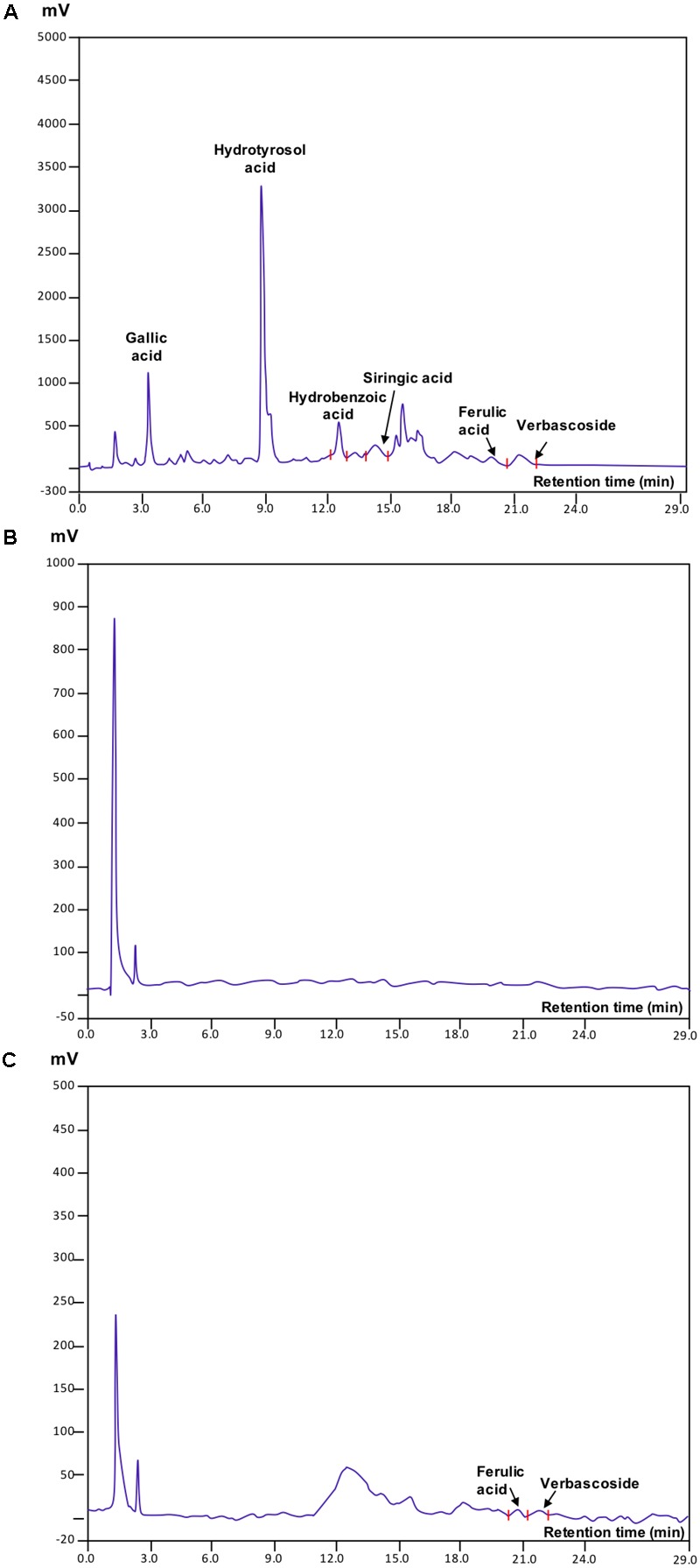
HPLC chromatograms of **(A)** olive mill wastewaters (OMW), and **(B)** control amendment (Amd-C) and **(C)** olive mill water filter plus MSW pre-treated organic material (OMWF) amendment after 6 months of OMW bio-oxidation.

The chromatographic profiles of OMW revealed the presence of multiple peaks corresponding to the following phenolic acids: gallic, hydroxytyrosol, hydroxybenzoic, syringic, ferulic, and verbacoside (**Figure 2A**). Among them, hydroxytyrosol acid was the most abundant. The analysis of extracts from control (Amd-C) (**Figure 2B**) and OMWF (**Figure 2C**) amendments confirmed the capacity of the bio-oxidation process to reduce the content of polyphenols within a short retention time (29 min) in the original OMW. Ferulic acid and verbascoside acid were still present in the OMWF amendment although in little amounts as in OMW, while the same compounds were missing in Amd-C. Quantification of individual phenols identified is reported in **Table 3**. Phytotoxicity tests via germination assays verified the good quality of OMW treated amendment (data not shown).

**Table 3:** Content (mg mL^-1^) of individual phenolic acids identified in olive mill wastewaters (OMW) before the bio-oxidation process, and in the obtained OMWF amendment.

Identified phenolic compound	OMW	OMWF
Gallic acid	23.62 ± 2.35	<LOD
Hydroxytyrosol acid	373.22 ± 3.98	<LOD
Hydroxybenzoic acid	30.65 ± 3.24	<LOD
Syringic acid	22.60 ± 2.11	<LOD
Ferulic acid	2.73 ± 0.45	0.07 ± 0.01
Verbascoside	17.86 ± 1.10	1.48 ± 0.18

*LOD, limit of detection*.

### Fourier Transform Infrared (FT-IR) and Dynamic Light Scattering (DLS) of Humic Acids

**FIGURE 3:**
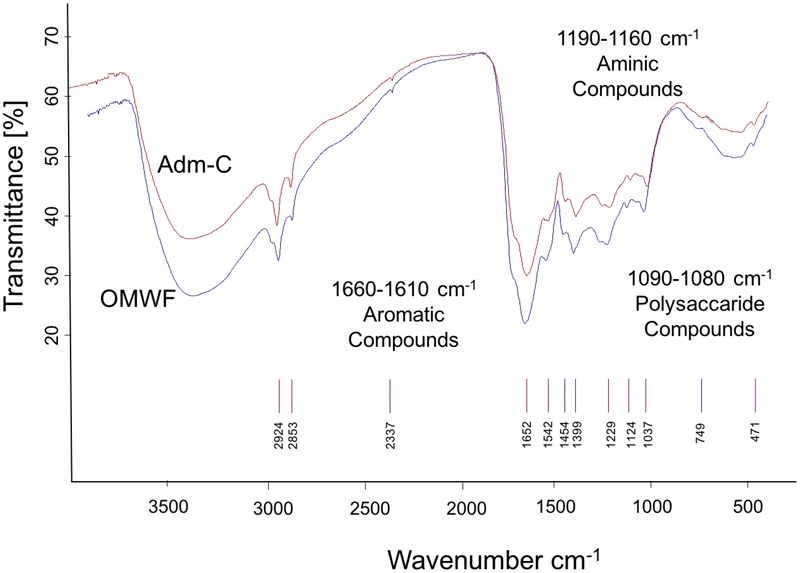
FT-IR spectra of humic acids (HA) extracted from control amendment (Amd-C) and olive mill water filter plus MSW pre-treated OMWF.

The OMWF amendment showed an important enrichment in humic acids (HA) as compared to Amd-C amendment. This result clearly indicates that the amount of total polyphenols in OMW was involved in the generation of humic acids during the bio-oxidative process. FT–IR spectra of HA of Amd-C and OMWF (**Figure 3**) showed bands of absorption corresponding to the major classes of organic compounds typical of HSs (AitBaddi et al., 2003). Indeed, they were characterized by the presence of strong bands attributed to carboxylic acid groups (1710–1715 cm^-1^), and bands associated to aromatic (1660–1610 cm^-1^), polysaccharide (1083 cm^-1^) and aminic compounds (1560–1590 cm^-1^) (Rao, 1963; Silverstein et al., 1981; Schulz and Baranska, 2007).

The peak at 1360–1580 cm^-1^ was attributed to the stretching of groups (COO-) of carboxylic compounds. In the amendment OMWF, a peak was visible at about 1170–1180 cm^-1^, which could be attributed to C–H vibrations of bending of the aromatic groups (Rao, 1963; Silverstein et al., 1981; Schulz and Baranska, 2007). In OMWF, an emission band very flared and irregular similar to a shoulder up to 2400 cm^-1^ was also evident and might indicate a different aromatic composition of the sample (Rao, 1963; Silverstein et al., 1981; Schulz and Baranska, 2007).

The particle sizes (D, diameter) and zeta potential (ZP) of HA obtained from Amd-C and OMWF amendments are reported in **Table 4**. Both HA data trends shared a similar behavior, which was characterized by a rapid decrease of particle size when the pH increased from 2 to 4. At higher pH values, the particle size of HA from Amd-C showed a systematic increment, while a weaker increase was observed for HA derived from OMWF. A similar trend was measured for zeta potential. In this case, HA from both amendments exhibited a neutral surface charge at low pH values. While increasing the pH, a progressive decrease in zeta potential was displayed by both series of HA data, which indicated a stronger colloidal stability of HA when the pH was neutral or basic.

**Table 4:** Diameter of the particles of humic acids and zeta potential at different pH values.

HA from Amd-C					HA from OMWF				

pH	*D* (nm)	PDI/D	ZP (mV)	IC (mS cm^-1^)	pH	*D* (nm)	PDI/D	ZP (mV)	IC (mS cm^-1^)
2	949 ± 45a	0.39 ± 0.03d	-2.68 ± 0.82c	15.10 ± 2.03a	2	869 ± 24a	0.41 ± 0.04c	-3.15 ± 0.23d	14.30 ± 1.40a
4	219 ± 34d	0.53 ± 0.06c	-27.90 ± 1.48b	1.81 ± 0.18b	4	313 ± 18d	0.47 ± 0.04c	-33.30 ± 1.52b	2.24 ± 0.24b
6	358 ± 40c	0.76 ± 0.07b	-30.80 ± 1.60ab	1.71 ± 0.20b	6	478 ± 26b	0.80 ± 0.06a	-27.10 ± 1.83c	2.31 ± 0.21b
8	424 ± 26c	0.70 ± 0.07b	-33.70 ± 1.23a	1.67 ± 0.33b	8	381 ± 25c	0.64 ± 0.06b	-30.66 ± 1.55bc	1.68 ± 0.23c
10	692 ± 40b	1.00 ± 0.08a	-34.90 ± 1.30a	2.02 ± 0.13b	10	419 ± 37bc	0.71 ± 0.03ab	-37.00 ± 2.10a	1.84 ± 0.14b

*D, diameter; PDI, Poly Dispersity Index; ZP, zeta potential; IC, ionic conductivity. Different letters along columns indicate significant differences (p < 0.05, ±SD, n = 3) according to Student–Newman–Keuls test.*

### Effects of HA From Amd-C and OMWF on Maize Plant Growth and Nutrient Content

**FIGURE 4:**
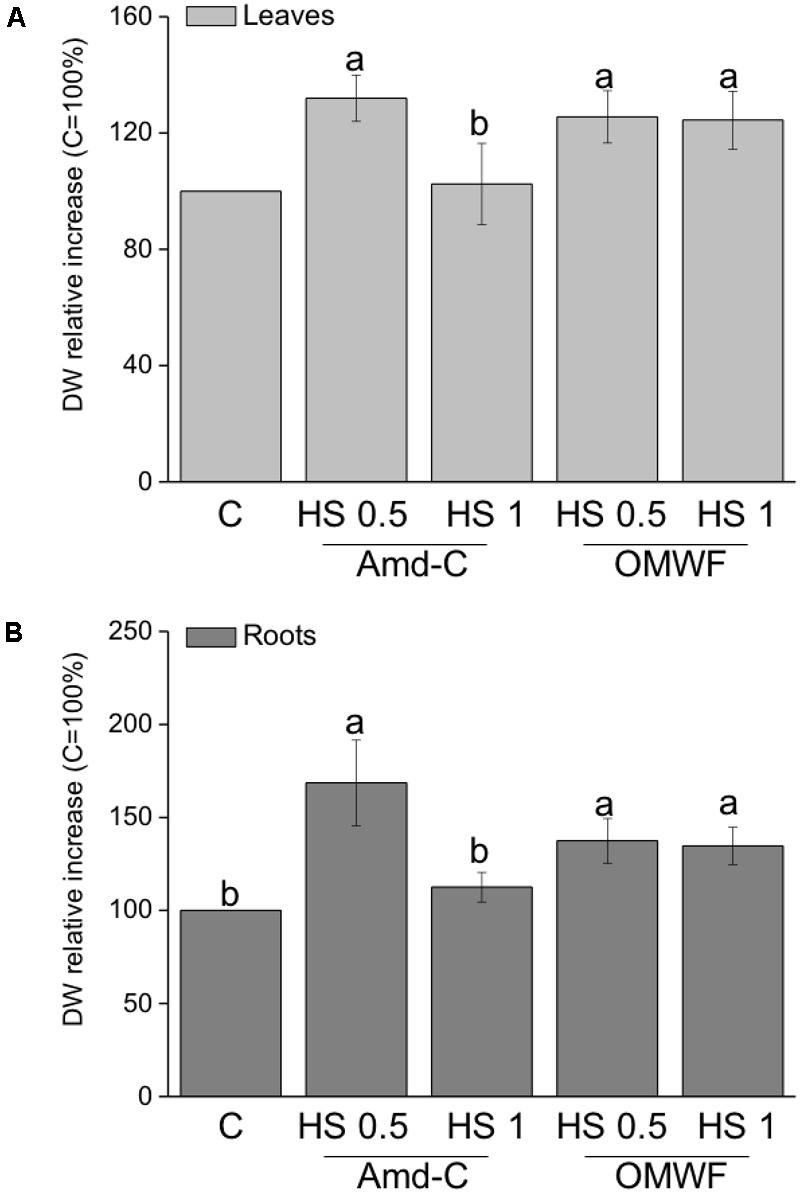
**(A)** Leaf and **(B)** root dry weight of *Zea mays* plants grown for 12 days in Hoagland modified nutrient solution and supplied for 2 days with HA extracted from control amendment (Amd-C) and olive mill water filter plus MSW pre-treated OMWF at 0.5 or 1 mg C L^-1^. Data represent the means of three measurements with five plants in each (±*SD*). Values are reported as percentage of leaf and root dry weight variation relative to the control (=100%). Different letters above bars indicate significant differences between treatments at *P* < 0.05 according to Student–Newman–Keuls test.

The effect of HA extracted from Amd-C and OMWF amendments on maize plant productivity was evaluated in terms of leaf and root dry biomass promotion (**Figures 4A,B**) and nutrient content (**Table 5**). The trend of growth stimulation for leaves was the same as for roots. HA extracted from Amd-C and supplied to plants at 0.5 mg C L^-1^ increased the dry weight (DW) of leaves and roots by about 32 and 68%, respectively, while at higher dosage (1 mg C L^-1^) they did not produce any significant change in biomass. Conversely, both dosages of HA derived from OMWF amendment determined similar increases in leaf and root growth (plus 35–37%), with values comparable to those measured for plants treated with HA from Amd-C at 0.5 mg C L^-1^.

**Table 5:** Leaf elemental composition of control maize plants (C, untreated) and plants supplied for 48 h with either HA derived from the amendment control (Amd-C) or HA obtained from amendment OMWF at two dosages (0.5 and 1 mg C L^-1^).

		Ca	K	Mg	B	Zn	Mn	Cu	Na
Leaves	C	7.66 ± 0.32b	41.61 ± 1.00b	4.24 ± 0.21b	14.1 ± 0.4c	91.9 ± 20.5b	66.2 ± 1.5b	13.3 ± 2.0c	191.7 ± 16.0a
Amd-C	HA 0.5	9.05 ± 0.26a	45.67 ± 1.51a	5.69 ± 0.28a	15.01 ± 0.3b	163.2 ± 15.1a	63.6 ± 2.8b	24.2 ± 3.5b	135.7 ± 17.5b
	HA 1.0	7.90 ± 0.28b	44.40 ± 1.40a	4.58 ± 0.39b	16.19 ± 0.2a	63.6 ± 19.9b	74.9 ± 2.6a	69.8 ± 6.7a	165.5 ± 9.0b
OMWF	HA 0.5	8.75 ± 0.29a	44.01 ± 1.16a	5.11 ± 0.24a	16.07 ± 0.6a	145.8 ± 15.4a	66.8 ± 1.8b	14.3 ± 2.8c	147.2 ± 34.3b
	HA 1.0	7.43 ± 0.34b	39.70 ± 3.29b	4.07 ± 0.31b	14.55 ± 0.6bc	99.5 ± 18.6b	72.3 ± 2.3a	9.9 ± 5.1c	144.6 ± 17.6b
Root	C	5.17 ± 0.39b	15.23 ± 0.30c	4.53 ± 0.30c	30.9 ± 1.8c	344.0 ± 6.3c	265.1 ± 15.7c	262.7 ± 19.8b	68.4 ± 11.2ab
Amd-C	HA 0.5	6.33 ± 0.28a	27.35 ± 1.70a	6.47 ± 0.68a	35.2 ± 2.7c	442.9 ± 25.4a	450.2 ± 27.6a	296.8 ± 25.5b	80.7 ± 11.4a
	HA 1.0	6.47 ± 0.29a	20.30 ± 1.43b	5.90 ± 0.47ab	31.4 ± 2.0c	431.3 ± 16.5a	324.7 ± 16.6b	226.4 ± 33.6b	88.9 ± 10.3a
OMWF	HA 0.5	5.08 ± 0.38b	20.76 ± 1.32b	5.26 ± 0.34b	47.8 ± 3.2a	361.9 ± 8.2b	339.6 ± 28.3b	254.5 ± 31.0b	59.7 ± 11.6b
	HA 1.0	3.69 ± 1.74b	19.24 ± 2.11b	4.45 ± 0.30c	38.5 ± 2.6b	117.6 ± 21.4d	385.1 ± 25.4b	489.2 ± 42.6a	48.0 ± 15.1b

*Values are expressed in mg g-^1^ d.wt. for macroelements, and in mg kg-^1^ d.wt. for microelements. Data represent the means of five measurements per treatment (±SD). Different letters along the same column indicate significant differences between treatments (p < 0.05) according to Student–Newman–Keuls test.*

With respect to the content in mineral nutrients, plants supplied with HA from Amd-C contained higher concentration of macro- and micro-elements in both leaves and roots compared to the untreated plants (**Table 5**). HA extracted from OMWF amendment exerted a general positive effect on nutrient accumulation when furnished to plants at lower dosage (0.5 mg C L^-1^), while they were almost ineffective at higher dosage (1 mg C L^-1^), as they only increased Mn in leaves and Cu in roots. The concentration of Na in plant tissues was also determined to evaluate whether Na contained in OMW could have been delivered to plants. Interestingly, we found a decrease of Na in leaves of maize plants supplied with HA from both amendments, while in roots Na reduction only occurred when plants were treated with HA from OMWF.

### Effects of HA From Amd-C and OMWF on Enzyme Activity and Protein Content

**FIGURE 5:**
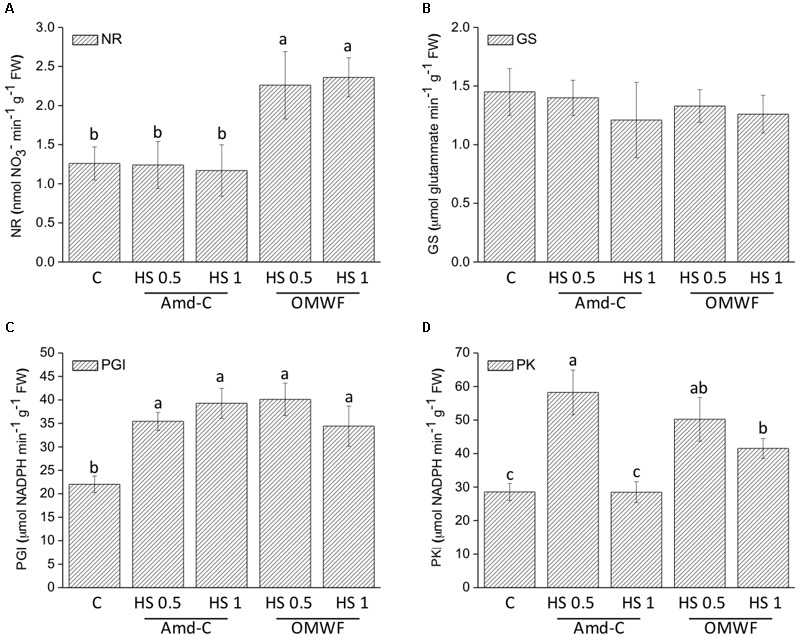
Effect of HA extracted from control amendment (Amd-C) and olive mill water filter plus MSW pre-treated OMWF on the activity of **(A)** nitrate reductase (NR), **(B)** glutamine synthase (GS), **(C)** phosphoglucose isomerase (PGI), and **(D)** pyruvate kinase (PK) enzymes in *Z. mays* plants grown for 12 days in a Hoagland modified nutrient solution and supplied for 2 days with humic acids (HA) extracted Amd-C and OMWF amendments at 0.5 or 1 mg C L^-1^. Data represent the means of three measurements with five plants in each (±*SD*). Different letters above bars indicate significant differences between treatments at *P* < 0.05 according to Student–Newman–Keuls test.

To assay the effects of HA on plant metabolism, changes in activity of enzymes working in glycolysis and nitrogen assimilation was evaluated in maize plants treated with HA derived from Amd-C and OMWF amendments (**Figures 5A–D**). The activity of NR, GS, PGI, and PK enzymes was differentially regulated by HA from Amd-C and OMWF amendments. Specifically, the activity of NR was significantly and equally enhanced by both dosages of HA extracted from OMWF being about 1.8–1.9-fold higher than the control, but no variation was observed between untreated plants and plants supplied with HA from Amd-C. GS activity was unchanged following HA application to plants, while PGI activity was strongly and similarly up-regulated by HA, regardless of the amendment origin and dosage. PK activity in plants supplied with both dosages of HA from OMWF or with 0.5 mg C L^-1^ of HA produced from Amd-C was higher than in control plants.

**FIGURE 6:**
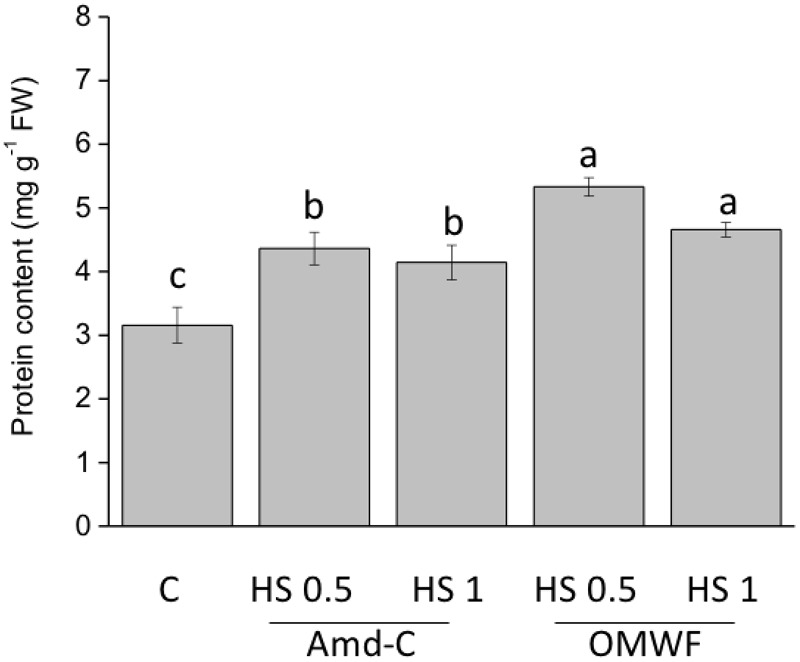
Effect of HA extracted from control amendment (Amd-C) and olive mill water filter plus MSW pre-treated OMWF on total leaf protein content in *Z. mays* plants grown for 12 days in a Hoagland modified nutrient solution and supplied for 2 days with HA extracted Amd-C and OMWF amendments at 0.5 or 1 mg C L^-1^. Data represent the means of three measurements (±*SD*). Different letters above bars indicate significant differences between treatments at *P* < 0.05 according to Student–Newman–Keuls test.

The two amendments increased the amount of total proteins at both dosages (**Figure 6**). However, the HA obtained from OMWF determined a more pronounced accumulation of proteins.

## Discussion

Findings obtained in this study indicate that humic acids extracted from an amendment obtained combining OMWs with a pre-treated organic material derived from MSW can be used as valuable biostimulants in agricultural practices by virtue of their positive effects on plant biomass production, nutrition and activity of enzymes implied in N metabolism and glycolysis. In support of our statement, previous work showed that biostimulants like HS can elicit morphological changes in plants, primarily a significant development of root biomass and the stimulation of root hair formation, which result in increased plant nutrient uptake and accumulation (Zandonadi et al., 2007, 2010; Nardi et al., 2016; Colla et al., 2017; Rouphael et al., 2017). In addition, HS and other types of biostimulants have been reported to up-regulate the gene expression and activity of enzymes catalyzing key steps of N assimilation and cell respiration processes due to their content in signaling molecules, such as amino acids, peptides, hormone-like substances and phenols (Crawford and Arst, 1993; Hoff et al., 1994; Schiavon et al., 2008; Ertani et al., 2009, 2013, 2014, 2017; Baglieri et al., 2014).

With respect to phenolic compounds in particular, a number of studies has shown that these molecules at low concentrations can trigger positive metabolic and physiological responses in plants (Ertani et al., 2011, 2018). Conversely, at concentrations as high as those normally recorded in OMW, phenols may be responsible for inhibition of soil microbiome activity and induction of several phytotoxic effects, including reduced seed germination, plant growth impairment and drops in productivity (Aggelis et al., 2003; Leonardis et al., 2009; Karpouzas et al., 2010; Leopoldini et al., 2011; García-Sánchez et al., 2012).

The phenolic composition of OMW varies depending on the climate, cultivar type and age, olives processing. Though, the most biologically active phenols commonly occurring in OMW are hydroxybenzoic acid, hydroxytyrosol acid, gallic acid, syringic acid, ferulic acid, caffeic acid, 3,4,5 trimethoxybenzoic acid, 3,4,5 trimethoxyphenyl-acetic acid, verbascoside, tyrosol acid, cyanidin, quercetin, and flavonols (Borja et al., 2006). Similarly to other OMW produced in Italy, the OMW used in this study contained elevate amounts of hydroxytyrosol acid and detectable levels of gallic acid, hydroxybenzoic acid, syringic acid, ferulic acid, and verbacoside (De Marco et al., 2007). These phenolic acids could cause toxicity to plants and soil microorganisms if OMW were spread on soil before being detoxified. However, their content significantly decreased after OMW composting with MSW. Also, based on results retrieved from germination assays (data not shown), the amendment obtained did not display phytotoxic effects. Therefore, our study confirms the effectiveness of the bio-oxidative treatment in reducing the hazardousness of OMW for potential recycling and application in agriculture.

The humic acids produced in the composting process and extracted from control and OMWF amendments were all endowed with a negative superficial charge (at neutral pH) and a particle size close to 500 nm. Spectroscopic analyses highlighted their pronounced aromatic features, with a low proportion of saturated aliphatic hydrocarbons (Colombo et al., 2015). Important differences in structural characteristics between HA from Amd-C and OMWF amendments were evidenced via DLS, which could be ascribed to differences in hydrophobicity and aromaticity of HA observed at pH 6. It is conceivable that at such pH value, HA extracted from OMWF amendment quickly formed micelle-like aggregates because of hydrophobic interactions of aromatic hydrocarbons between particles (Angelico et al., 2014). In support of this hypothesis, HA from OMWF showed a higher particle size (500 nm) at pH 6. On the other hand, the size of HA particles from Amd-C was lower (<360 nm) at the same pH, likely due to intermolecular electrostatic repulsion between the acidic functional groups. The elongate shape of both HA was suggested by the high size observed at pH < 2 (Colombo et al., 2015). The small but significant differences in size and shape between HA from Amd-Control and OMWF could be explained by OMW contribution to the aromatic chemical structures during the bio-oxidative process.

Differences in structural properties between HA obtained from Amd-Control and OMWF could justify the differential their effects on plant biomass, activity of marker enzymes and accumulation of several nutrients. HA have been reported to stimulate plant growth by targeting pivotal steps of plant metabolism, especially N assimilation and cell respiration (Nardi et al., 2007). In this study, HA from Amd-Control enhanced leaf and root biomass of maize plants when supplied at lower dosage (0.5 mg C L^-1^), but they did not influence the activity of NR and GS enzymes, which catalyze the reduction of nitrate to nitrite and the synthesis of the amino acid glutamine, respectively. However, these HA increased the activity of key enzymes functioning in glycolysis, i.e., PGI at both dosages and PK at lower dosage, thus indicating their capacity to increase cell respiration and induce higher production of ATP molecules to be used for energy-dependent cellular work. HA extracted from OMWF promoted plant biomass production at both dosages, and significantly increased the activity of NR, PGI and PK. Therefore, N assimilation and cell respiration were both metabolic targets of these HA, likely because of their chemical and structural properties, such as prominent hydrophobicity and aromaticity (Jindo et al., 2012; Martinez-Balmori et al., 2014). It is also possible that some compounds contained in detoxified OMW and further included in the HA structure acted as signaling molecules in plants, thus leading to higher activity of both N and C enzymes. The more pronounced effect of HA from OMWF on N assimilation was confirmed by values of protein accumulation in plants.

Treating maize plants with HA extracted from the two amendments promoted nutrient accumulation in plant tissues. This effect was previously reported for other HS and biostimulants in several studies, and has been related to their capacity to stimulate root growth and development, root hair formation, expression of nutrient transporters and activity of plasma membrane H^+^-ATP-ase (Zandonadi et al., 2007, 2010; Ertani et al., 2013, 2017; Nardi et al., 2016; Santi et al., 2017). The lower capacity of HA derived from OMWF to enhance plant nutrition at higher dosage (1 mg C L^-1^) could be due to the presence of one or more undetermined compounds derived from OMW, which could have modified the root cell membrane permeability, thus preventing the increase of nutrient uptake mediated by HA, despite both ATP synthesis and root growth were stimulated.

## Conclusion

This study confirms the effectiveness of OMW bio-oxidation with a pre-treated organic material derived from MSW in decreasing the phenolic loading of OMW and producing stabilized organic amendments, in line with the legislative parameters of mixed organic amendments (absence of pathogens, heavy metal concentrations lower the threshold toxicity, no phytotoxicity effects). In addition, humic acids extracted from OMWF amendment could be used as valuable biostimulants in agriculture practices as evinced by their capacity to promote plant growth, activity of marker enzyme and nutrient accumulation significantly.

## Author Contributions

GP performed the chemical analyses on OMW, MSW, HA, and nutrients in plants. MS wrote the manuscript. SN edited the manuscript. AE performed the analyses on plants and helped in writing the manuscript. GC and CC designed and supervised the experiments.

## Conflict of Interest Statement

The authors declare that the research was conducted in the absence of any commercial or financial relationships that could be construed as a potential conflict of interest.

## References

[B1] Adani F. Genevini P. L. Gasperi F. Zorzi G. (1997). Organic matter evolution index (OMEI) as a measure of amendment inefficiency. *Compost Sci. Util.* 5 53–62. 10.1080/1065657X.1997.10701874

[B2] Aggelis G. Iconomou D. Christou M. Bokas D. Kotzailias S. Christou G. (2003). Phenolic removal in a model olive oil mill wastewater using Pleurotusostreatus. *Water Res.* 16 3897–3904. 10.1016/S0043-1354(03)00313-0 12909108

[B3] Ahmadi-Esfahani F. Z. (2006). Constant market shares analysis, uses, limitations and prospects. *Aust. J. Agric. Resour. Econ.* 50 510–526. 10.1111/j.1467-8489.2006.00364.x

[B4] AitBaddi G. Hafidi M. Gilard V. Revel J. C. (2003). Characterization of humic acids produced during composting of olive mill wastes: elemental and spectroscopic analyses (FTIR and 13C NMR). *Agronomie* 23 661–666. 10.1051/agro:2003042

[B5] Andreozzi R. Canterino M. Di-Somma I. Lo-Giudice R. Marotta R. Pinto G. (2008). Effect of combined physico-chemical processes on the phytotoxicity of olive mill wastewaters. *Water Res.* 42 1684–1692. 10.1016/j.watres.2007.10.018 18006039

[B6] Andreozzi R. Longo G. Majone M. Modesti G. (1998). Integrated treatment of olive oil mill effluents (OME): study of ozonation coupled with anaerobic digestion. *Water Res.* 32 2357–2364. 10.1016/S0043-1354(97)00440-5

[B7] Angelico R. Ceglie A. Ji-Zheng J. Liu-Rong L. Palumbo G. Colombo C. (2014). Particle size, charge and colloidal stability of humic acids coprecipitated with Ferrihydrite. *Chemosphere* 99 239–247. 10.1016/j.chemosphere.2013.10.092 24315181

[B8] Arvanitoyannis I. S. Kassaveti A. Stefanatos S. (2007). Olive oil waste treatment: a comparative and critical presentation of methods, advantages & disadvantages. *Crit. Rev. Food Sci. Nutr.* 47 187–229. 10.1080/10408390600695300 17453921

[B9] Baglieri A. Cadili V. Mozzetti Monterumici C. Gennari M. Tabasso S. Montoneri E. (2014). Fertilization of bean plants with tomato plants hydrolysates. Effect on biomass production, chlorophyll content and N assimilation. *Sci. Hortic.* 176 194–199. 10.1016/j.scienta.2014.07.002

[B10] Bonadonna L. Briancesco R. Chiaretti G. Coccia A. M. Della Libera S. Marini R. (2002). *Microbiological Quality of Composting Products: Legislative and Hygienic Measures. Operative Procedures for the Microbiological Analytical Control.* Rome: Istituto Superiore di Sanità.

[B11] Borja R. Sànchez E. Raposo F. Rincòn B. Jiménez A. M. Martìn A. (2006). Study of the natural biodegradation of two-phase olive mill solid waste during its storage in an evaporation pond. *Waste Manag.* 26 477–486. 10.1016/j.wasman.2005.02.024 15963711

[B12] Bradford M. (1976). A rapid and sensitive method for the quantitation of microgram quantities of protein utilizing the principle of protein-dye binding. *Anal. Biochem.* 1976 248–254. 10.1016/0003-2697(76)90527-3 942051

[B13] Bremner J. M. Keeney D. R. (1965). Steam distillation methods for determination of ammonium, nitrate and nitrite. *Anal. Chim. Acta* 32 485–495. 10.1016/S0003-2670(00)88973-4

[B14] Canellas L. P. Olivares F. L. Okorokova-Facanha A. L. Facanha A. R. (2002). Humic acids isolated from earthworm amendment enhance root elongations, lateral root emergence, and plasma membrane H+-ATPase activity in maize roots. *Plant Physiol.* 130 1951–1957. 10.1104/pp.007088 12481077 PMC166705

[B15] Casa R. D’Annibale A. Pieruccetti F. Stazi S. R. Giovannozzi S. LoCascio B. (2003). Reduction of the phenolic components in olive-millwaste water by enzymatic treatment and its impact on durum wheat (*Triticum durum* Desf.) germinability. *Chemosphere* 50 959–966. 10.1016/S0045-6535(02)00707-5 12531700

[B16] Colarieti M. L. Toscano G. Greco G. (2006). Toxicity attenuation of olive mill wastewater in soil slurries. *Environ. Chem. Lett.* 4 115–118. 10.1007/s10311-006-0050-5

[B17] Colla G. Hoagland L. Ruzzi M. Cardarelli M. Bonini P. Canaguier R. (2017). Biostimulant action of protein hydrolysates: unraveling their effects on plant physiology and microbiome. *Front. Plant Sci.* 8:2202. 10.3389/fpls.2017.02202 29312427 PMC5744479

[B18] Colombo C. Palumbo G. Angelico R. Cho H. G. Francioso O. Ertani A. (2015). Spontaneous aggregation of humic acid observed with AFM at different pH. *Chemosphere* 138 821–828. 10.1016/j.chemosphere.2015.08.010 26295541

[B19] Crawford N. M. Arst H. N. Jr. (1993). The molecular genetics of nitrate assimilation in fungi and plants. *Annu. Rev. Genet.* 27 115–146. 10.1146/annurev.ge.27.120193.000555 8122899

[B20] De Marco E. Savarese M. Paduano A. Sacchi R. (2007). Characterization and fractionation of phenolic compounds extracted from olive oil mill wastewaters. *Food Chem.* 104 858–867. 10.1016/j.foodchem.2006.10.005

[B21] Di Serio M. G. Lanza B. Mucciarella M. R. Russi F. Iannucci E. Marfisi P. (2008). Effects of olive mill wastewater spreading. *Int. Biodet. Biodeg.* 62 403–407. 10.1016/j.ibiod.2008.03.006

[B22] European Biostimulants Industry Council [EBIC] (2013). *Economic Overview of the Biostimulants Sector in Europe.* Available at: http://www.biostimulants.eu

[B23] El-Gohary F. Tawfik A. Badawy M. El-Khateeb M. A. (2009). Potentials of anaerobic treatment for catalytically oxidized olive mill wastewater (OMW). *Bioresour. Technol.* 100 2147–2154. 10.1016/j.biortech.2008.10.051 19070481

[B24] Ertani A. Cavani L. Pizzeghello D. Brandellero E. Altissimo A. Ciavatta C. (2009). Biostimulant activity of two protein hydrolysates on the growth and nitrogen metabolism in maize seedlings. *J. Plant Nutr. Soil Sci.* 172 237–244. 10.1002/jpln.200800174

[B25] Ertani A. Francioso O. Tinti A. Schiavon M. Pizzeghello D. Nardi S. (2018). Evaluation of seaweed extracts from *Laminaria* and *Ascophyllum nodosum* spp. as biostimulants in *Zea mays* L. using a combination of chemical, biochemical and morphological approaches. *Front. Plant Sci.* 9:428. 10.3389/fpls.2018.00428 29681909 PMC5897654

[B26] Ertani A. Pizzeghello D. Baglieri A. Cadili V. Tambone F. Gennari M. (2013). Humic-like substances from agro-industrial residues affect growth and nitrogen assimilation in maize (*Zea mays* L.) plantlets. *J. Geochem. Exp.* 129 103–111. 10.1016/j.gexplo.2012.10.001

[B27] Ertani A. Pizzeghello D. Francioso O. Sambo P. Sanchez-Cortes S. Nardi S. (2014). *Capsicum chinensis* L. growth and nutraceutical properties are enhanced by biostimulants in a long-term period: chemical and metabolomic approaches. *Front. Plant. Sci.* 5:375. 10.3389/fpls.2014.00375 25136346 PMC4117981

[B28] Ertani A. Schiavon M. Altissimo A. Franceschi C. Nardi S. (2011). Phenol-containing organic substances stimulate phenylpropanoid metabolism in *Zea mays*. *J. Plant Nutr. Soil Sci.* 174 496–503. 10.1002/jpln.201000075

[B29] Ertani A. Schiavon M. Nardi S. (2017). Transcriptome-wide identification of differentially expressed genes in *Solanum lycopersicon* L. in response to an alfalfa-protein hydrolysate using microarrays. *Front. Plant Sci.* 8:1159. 10.3389/fpls.2017.01159 28725232 PMC5496959

[B30] Eyheraguibel B. Silvestre J. Morard P. (2008). Effects of humic substances derived from organic waste enhancement on the growth and mineral nutrition of maize. *Bioresour. Technol.* 99 4206–4212. 10.1016/j.biortech.2007.08.082. 17962015

[B31] Flores N. Brillas E. Centellas F. Rodríguez R. M. Cabot P. L. Garrido J. A. (2018). Treatment of olive oil mill wastewater by single electrocoagulation with different electrodes, and sequential electrocoagulation/electrochemical Fenton-based processes. *J. Hazard. Mater.* 5 58–66. 10.1016/j.jhazmat.2017.12.059 29289766

[B32] García-Sánchez M. Garrido I. Casimiro I. J. Casero P. J. Espinosa F. García-Romera I. (2012). Defence response of tomato seedlings to oxidative stress induced by phenolic compounds from dry olive mill residue. *Chemosphere* 89 708–716. 10.1016/j.chemosphere.2012.06.026 22818883

[B33] Ghanbari R. Anwar F. Alkharfy K. M. Gilani A. H. Saari N. (2012). Valuable nutrients and functional bioactives in different parts of olive (*Olea europaea* L.) – a review. *Int. J. Mol. Sci.* 13 3291–3340. 10.3390/ijms13033291 22489153 PMC3317714

[B34] Gigliotti G. Proietti P. Said-Pullicino D. Nasini L. Pezzolla D. (2012). Co-amendmenting of olive husks with high moisture contents: organic matter dynamics and amendment quality. *Int. Biodeter. Biodegr.* 67 8–14. 10.1016/j.ibiod.2011.11.009

[B35] Hamdi M. Khadir A. Garcia J. L. (1991). The use of *Aspergillus niger* for bioconversion of olive mill wastewater. *Appl. Microbiol. Biotechnol.* 34 828–831. 10.1007/BF00169359

[B36] Hoagland D. R. Arnon D. I. (1950). The water-culture method for growing plants without soil. *Calif. Agric. Exp. Stn. Circ.* 347 1–32.

[B37] Hoff T. Truong H. N. Caboche M. (1994). The use of mutants and transgenic plants to study nitrate assimilation. *Plant Cell Environ.* 17 489–506. 10.1111/j.1365-3040.1994.tb00145.x

[B38] Incelli M. Cordiner S. Tosti S. Borgognoni F. Sansovini M. Santucci A. (2016). Trattamento delle acque di vegetazione do oleifici: tecnologie a membrana e processi termochimici. *Chim. Ind.* 3 10.17374/CI.2016.3.4.1

[B39] International Olive Council (2014). *Oil Organization, “World Olive Oil Figures”.* Available at: http://www.internationaloliveoil.org/estaticos/view/131-world-olive-oil-figures

[B40] Isidori M. Lavorgna M. Nardelli A. Parrella A. (2005). Model study on the effect of 15 phenolic olive mill wastewater constituents on seed germination and vibrofischeri metabolism. *J. Agric. Food Chem.* 53 8414–8417. 10.1021/jf0511695 16218695

[B41] Jindo K. Martim S. A. Navarro E. C. Pérez-Alfocea F. Hernandez T. Garcia C. (2012). Root growth promotion by humic acids from composted and non-composted urban organic wastes. *Plant Soil* 353 209–220. 10.1007/s11104-011-1024-3

[B42] Justino C. I. Pereira R. Freitas A. C. Rocha-Santos T. A. Panteleitchouk T. S. Duarte A. C. (2012). Olive oil mill wastewaters before and after treatment: a critical review from the ecotoxicological point of view. *Ecotoxicology* 21 615–629. 10.1007/s10646-011-0806-y 22042608

[B43] Karpouzas D. G. Ntougias S. Iskidou E. Rousidou C. Papadopoulou K. K. Zervakis G. G. (2010). Olive mill wastewater affects the structure of soil bacterial communities,”. *Appl. Soil Ecol.* 45 101–111. 10.1016/j.apsoil.2010.03.002

[B44] Khoufi S. Aloui F. Sayadi S. (2008). Extraction of antioxidants from olive mill wastewater and electro-coagulation of exhausted fraction to reduce its toxicity on anaerobic digestion. *J. Hazard. Mater.* 151 531–539. 10.1016/j.jhazmat.2007.06.017 17629620

[B45] Komilis D. P. Karatzas E. Halvadakis C. P. (2005). The effect of olive mill wastewater on seed germination after various pre-treatment techniques. *J. Environ. Manage.* 74 339–348. 10.1016/j.jenvman.2004.09.009 15737458

[B46] Koutsos T. M. Chatzistathis T. Balampekou E. I. (2018). A new framework proposal, towards a common EU agricultural policy, with the best sustainable practices for the re-use of olive mill wastewater. *Sci Total Environ.* 62 942–953. 10.1016/j.scitotenv.2017.12.073 29227945

[B47] Leonardis A. Macciola V. Nag A. (2009). Antioxidant activity of various phenolextracts of olive-oil mill wastewaters. *Acta Alim.* 38 77–86. 10.1556/AAlim.2008.0030

[B48] Leopoldini M. Russo N. Toscano M. (2011). The molecular basis of working mechanism of natural polyphenolic antioxidants. *Food Chem.* 125 288–306. 10.1016/j.foodchem.2010.08.012

[B49] Lewis O. A. M. Watson E. F. Hewitt E. J. (1982). Determination of nitrate reductase activity in barley leaves and roots. *Ann. Bot.* 49 31–37. 10.1093/oxfordjournals.aob.a086227

[B50] Martinez-Balmori D. Spaccini R. Aguiar N. O. Novotny E. H. Olivares F. L. Canellas L. P. (2014). Molecular characteristics of humic acids isolated from vermicomposts and their relationship to bioactivity. *J. Agric. Food Chem.* 62 11412–11419. 10.1021/jf504629c 25379603

[B51] Mechri B. Echbili A. Issaoui M. Braham M. Elhadj S. B. Hammami M. (2007). Short-term effects in soil microbial community following agronomic application of olive mill wastewaters. *Appl. Soil Ecol.* 36 216–223. 10.1080/03601234.2013.742398 23431977

[B52] Mekki A. Dhouib A. Sayadi S. (2006). Changes in microbial and soil properties following amendment with treated and untreated olive mill wastewater. *Microbiol. Res.* 161 93–101. 10.1016/j.micres.2005.06.001 16427511

[B53] Mora V. Bacaicoa E. Zamarreño A. M. Aguirre E. Garnica M. Fuentes M. (2010). Action of humic acid on promotion of cucumber shoot growth involves nitrate-related changes associated with the root-to-shoot distribution of cytokinins, polyamines and mineral nutrients. *J. Plant Physiol.* 167 633–642. 10.1016/j.envexpbot.2011.10.001 20185204

[B54] Muscolo A. Sidari M. Nardi S. (2013). Humic substance: relationship between structure and activity. Deeper information suggests univocal findings. *J. Geochem. Explor.* 129 57–63. 10.1016/j.gexplo.2012.10.012

[B55] Nardi S. Concheri G. Dell’Agnola G. Scrimin P. (1991). Nitrate uptake and ATPase activity in oat seedlings in the presence of two humic fractions. *Soil Biol. Biochem.* 23 833–836. 10.1016/0038-0717(91)90094-Z

[B56] Nardi S. Ertani A. Francioso O. (2017). Soil–root cross-talking: the role of humic substances. *J. Plant Nutr. Soil Sci.* 180 5–13. 10.1002/jpln.201600348

[B57] Nardi S. Muscolo A. Vaccaro S. Baiano S. Spaccini R. Piccolo A. (2007). Relationship between molecular characteristics of soil humic fractions and glycolytic pathway and krebs cycle in maize seedlings. *Soil Biol. Biochem.* 39 3138–3146. 10.1016/j.soilbio.2007.07.006

[B58] Nardi S. Panucci M. R. Abenavoli M. R. Muscolo A. (1994). Auxin-like effect of humic substances extracted from faeces of *Allolobophora Caliginosa* and *Allolobophora Rosea*. *Soil Biol. Biochem.* 23 833–836. 10.1016/0038-0717(91)90094-Z

[B59] Nardi S. Pizzeghello D. Muscolo A. Vianello A. (2002). Review “physiological effects of humic substances on higher plants”. *Soil Biol. Biochem.* 32 1527–1536. 10.1016/S0038-0717(02)00174-8

[B60] Nardi S. Pizzeghello D. Schiavon M. Ertani A. (2016). Plant biostimulants: physiological responses induced by protein hydrolyzed-based products and humic substances in plant metabolism. *Sci. Agric.* 73 18–23. 10.1590/0103-9016-2015-0006

[B61] Nasini L. Gigliotti G. Balduccini M. A. Federici E. Cenci G. Proietti P. (2013). Effect of solid olive-mill waste amendment on soil fertility and olive (*Olea europaea* L.) tree activity,”. *Agric. Ecosyst. Environ.* 164 292–297. 10.1016/j.agee.2012.10.006

[B62] Niaounakis M. Halvadakis C. P. (2006). *Olive Processing Waste Management: Literature Review and Patent Survey* 2nd Edn Kidlington: Elsevier Ltd.

[B63] Nowotny I. Schwanz J. Rothe G. M. (1998). Influence of soil acidification and liming on selected enzymes of the carbohydrate metabolism and the contents of two major organic acids of mycorrhizal roots of Norway spruce (*Picea abies* [L.] Karst). *Plant Soil* 199 41–51. 10.1023/A:1004221910199

[B64] Ntougias S. Gaitis F. Katsaris P. Skoulika S. Iliopoulos N. Zervakis G. I. (2013). The effects of olives harvest period and production year on olive mill wastewater properties evaluation of pleurotus strains as bioindicators of the effluent’s toxicity. *Chemosphere* 92 399–405. 10.1016/j.chemosphere.2013.01.033 23399310

[B65] Ouzounidou G. Zervakis G. I. Gaitis F. (2010). Raw and microbiologically detoxified olive mill waste and their impact on plant growth. *Terr. Aquat. Environ. Toxicol.* 4 21–38.

[B66] Oztekin R. Sponza D. T. (2013). Treatment of wastewaters from the olive mill industry by sonication. *J. Chem. Technol. Biotechnol.* 88 212–225. 10.1002/jctb.3808

[B67] Paredes C. Bernal M. P. Cegarra J. Roig A. (2002). Bio-degradation of olive mill wastewater sludge by its co-amendmenting with agricultural wastes. *Bioresour. Technol.* 85 1–8. 10.1016/S0960-8524(02)00078-0 12146635

[B68] Piccolo A. (2001). The supramolecular structure of humic substances. *Soil Sci.* 166 810–832. 10.1097/00010694-200111000-00007

[B69] Rao C. N. R. (1963). *Chemical Application of Infrared Spectroscopy.* New York, NY: Science Academic Press.

[B70] Rinaldi M. Rana G. Introna M. (2003). Olive-mill wastewater spreading in southern Italy: effects on a durum wheat crop. *Field Crops Res.* 84 319–326. 10.1016/S0378-4290(03)00097-2

[B71] Roig A. Cayuela M. L. Sánchez-Monedero M. A. (2006). An overview on olive mill wastes and their valorisation methods. *Waste Manag.* 26 960–969. 10.1016/j.wasman.2005.07.024 16246541

[B72] Rouphael Y. Colla G. Giordano M. El-Nakhel C. Kyriacou M. C. De Pascale S. (2017). Foliar applications of a legume-derived protein hydrolysate elicit dose-dependent increases of growth, leaf mineral composition, yield and fruit quality in two greenhouse tomato cultivars. *Sci. Hortic.* 226 353–360. 10.1016/j.scienta.2017.09.007

[B73] Sampedro I. D’Annibale A. Ocampo J. A. Stazi S. R. Garcia Romera I. (2007). Solid-state cultures of *Fusarium oxysporum* transform aromatic components of olive-mill dry residue and reduce its phytotoxicity. *Bioresour. Technol.* 98 3547–3554. 10.1016/j.biortech.2006.11.015 17207620

[B74] Sanchez Monedero M. A. Cegarra J. Garcia D. Roing A. (2002). Chemical and structural evolution of humic acids during organic waste amendmenting. *Biodegradation* 13 361–371. 10.1023/A:1022888231982 12713128

[B75] Santi C. Zamboni A. Varanini Z. Pandolfini T. (2017). Growth stimulatory effects and genome-wide transcriptional changes produced by protein hydrolysates in maize seedlings. *Front. Plant Sci.* 8:433. 10.3389/fpls.2017.00433 28424716 PMC5371660

[B76] Schaumann G. E. (2006). Soil organic matter beyond molecular structure Part 1: macromolecular and supramolecular characteristics. *J. Plant. Nutr. Soil Sci.* 169 145–156. 10.1002/jpln.200521785

[B77] Schiavon M. Ertani A. Nardi S. (2008). Effects of an alfalfa protein hydrolysate on the gene expression and activity of enzymes of TCA cycle and N metabolism in *Zea mays* L. *J. Agric. Food Chem.* 56 11800–11808. 10.1021/jf802362g 19053364

[B78] Schulz H. Baranska M. (2007). Identification and quantification of valuable plant substances by IR and Raman spectroscopy. *Vib. Spectrosc.* 43 13–25. 10.1016/j.vibspec.2006.06.001 12696923

[B79] Sellami F. Jarboui R. Hachicha S. Medhioub K. Ammar E. (2008). Co-amendmenting of oil exhausted olive-cake, poultry manure and industrial residues of agro-food activity for soil amendment. *Bioresour. Technol.* 99 1177–1188. 10.1016/j.biortech.2008.01.055 17433668

[B80] Silva A. M. T. Nouli E. Xekoukoulotakis N. P. Mantzavinos D. (2007). Effect of key operating parameters on phenols degradation during H2O2-assisted TiO2 photocatalytic treatment of simulated and actual olive mill wastewaters. *Appl. Catal. B Environ.* 73 11–22. 10.1016/j.apcatb.2006.12.007

[B81] Silverstein R. M. Bassler G. C. Morrill T. C. (1981). *Spectrometric Identification of Organic Compounds* 4th Edn New York NY John Wiley and Sons.

[B82] Sokal R. R. Rohlf F. J. (1969). *Biometry: The Principles and Practice of Statistics in Biological Research.* San Francisco, CA: W. H. Freeman 776.

[B83] Trombetta A. Accotto E. Belfiore G. Piccone G. Pantusa S. Nappi P. (1998). *Metodi di Analisi dei Compost: Determinazioni Chimiche, Fisiche, Biologiche e Microbiologiche: Analisi Merceologiche dei Rifiuti, Volume 6 of Collana Ambiente.* Piemonte: Assessorato All’ambiente 1–187.

[B84] Vaccaro S. Muscolo A. Pizzeghello D. Spaccini R. Piccolo A. Nardi S. (2009). Effect of a compost and its water-soluble fractions on key enzymes of nitrogen metabolism in maize seedlings. *J. Agric. Food Chem.* 57 11267–11276. 10.1021/jf901808s 19891475

[B85] Varanini Z. Pinton R. (2001). “Directve MSWs indirect effects of soil humic substances on plant growth and nutrition,” in *The Rizosphere* eds Pinton R. Varanini Z. Nannipieri P. (Basel: Marcel Dekker) 141–158.

[B86] Violante P. (2000). *Metodi di Analisi Chimica del Suolo.* Milan: Franco Angeli.

[B87] Wershaw R. L. (1999). Molecular aggregation of humic substances. *Soil Sci.* 164 803–813. 10.1097/00010694-199911000-00004

[B88] Zandonadi D. B. Canellas L. P. Façanha A. R. (2007). Indolacetic and humic acids induce lateral root development through a concerted plasmalemma and tonoplast H+ pumps activation. *Planta* 225 1583–1595. 10.1007/s00425-006-0454-2 17180358

[B89] Zandonadi D. B. Santos M. P. Dobbss L. B. Olivares F. L. Canellas L. P. Binzel M. L. (2010). Nitric oxide mediates humic acids-induced root development and plasma membrane H+-ATPase activation. *Planta* 231 1025–1036. 10.1007/s00425-010-1106-0 20145950

[B90] Zullo B. A. Di Stefano M. G. Cioccia G. Ciafardini G. (2014). Evaluation of polyphenol decay in the oily fraction of olive fruit during storage using a mild sample handling method. *Eur. J. Lipid Sci. Technol.* 116 160–168. 10.1002/ejlt.201300287

